# The Hahnemannian’s Analysis Sheet

**Published:** 1891-07

**Authors:** Alfred Heath


### THE HAHNEMANNIAN'S ANALYSIS SHEET.

To the Editors of The Homœopathic Physician:

I had not the faintest intention of charging Dr. Wolff with plagiarism, and I am sorry he should have so misunderstood my letter. If he will read the "review" of his plan in the February Homœopathic Physician, and also the first part of my letter in the April number, he will see that *you say* it is a modification of Dr. E. J. Lee's plan, and of the *later device of Dr. Alfred Heath.* It was to this last that I took exception. I

could not personally say anything about Dr. Wolff's plan, as I have never seen it, so I must leave you to settle the point as to any similarity that may exist between Dr. Lee's and Dr. Wolff's plan. I do not know that there is any particular honor in proving "originality." It is a kind of plan that may occur to many men when working out a case. My only idea in publishing it was that it may not occur to all, as proved by the fact that it has been so much thought of, especially by beginners. It has also opened the eyes of many allopaths to the truth of the law of similars. They cannot get away from the fact that if the "working" points to a remedy (not even thought of), which *produces* all the symptoms of the patient, and that remedy cures the disease, Homœopathy is unquestionably a science, and that this science is founded on a natural law which in itself must be infallible. I am, dear sirs,

Yours truly,

ALFRED HEATH.

